# 1.5T MRI评估肺动脉高压患者右心功能及肺动脉血液动力学

**DOI:** 10.3779/j.issn.1009-3419.2012.08.04

**Published:** 2012-08-20

**Authors:** 艳 韩, 振文 杨, 铁链 于, 静 于, 米娜 薛, 璋 张, 东 李

**Affiliations:** 1 300052 天津，天津医科大学总医院放射科 Department of Radiology, Tianjin Medical University General Hospital, Tianjin 300052, China; 2 300052 天津，天津医科大学总医院心内科 Department of Cardiovascular Disease, Tianjin Medical University General Hospital, Tianjin 300052, China

**Keywords:** 磁共振成像, 高血压，肺性, 心室功能，右肺动脉, 血液动力学, Magnetic resonance imaging, Hypertension, pulmonary, Ventricular function, Right pulmonary artery, Hemodynamics

## Abstract

**背景与目的:**

肺动脉高压（pulmonary hypertension, PH）患者肺动脉压力增高，右心功能逐渐下降，最终导致右心衰竭而死亡，因而准确、无创地监测PH患者右心功能及肺动脉血液动力学改变尤为重要。本研究旨在评价心脏MRI（cardiac magnetic resonance imaging, CMRI）在评估PH患者右心功能及肺动脉血液动力学中的价值。

**方法:**

对25例PH患者及30例健康志愿者进行心脏电影MRI（cine-MRI）及相位对比法MRI（PC-MRI）扫描。对cine-MRI扫描图像进行后处理，计算获得右心室舒张末期容积（end-diastolic volume, EDV）、收缩末期容积（end-systolic volume, ESV）、搏出量（stroke volume, SV）、射血分数（ejection fraction, EF）及心肌质量（myocardial mass, MM），以上数据除EF外均经体表面积（body surface area, BSA）校正。对PC-MRI扫描图像进行处理并计算获得主肺动脉（main pulmonary artery, MPA）峰值流速及其顺应性。采用两独立样本*t*检验分析两组参数有无差异，明显性标准为*P* < 0.05。

**结果:**

与对照组相比，PH组右心室EDV、ESV及MM指数均明显高于后者（*P* < 0.01），EF明显低于后者（*P* < 0.01），SV指数与对照组无明显差异（*P* > 0.05），MPA峰值流速及顺应性均明显低于对照组（*P* < 0.01）。

**结论:**

CMRI在PH患者右心功能及MPA血液动力学参数评估中具有重要意义。

## Abstract (en)

**Background and objective:**

Pulmonary hypertension (PH) is characterized by rising pulmonary arterial pressure, decreasing right ventricular (RV) function, and ultimately, RV failure. Therefore, it is important to monitor RV function and pulmonary artery hemodynamics accurately and noninvasively. This study evaluates cardiac magnetic resonance imaging (CMRI) in assessing RV function and pulmonary artery hemodynamics in patients with PH.

**Methods:**

Cine-MRI and phase-contrast MRI (PC-MRI) were performed in 25 PH patients and 30 healthy volunteers. Cine-MRI images were post-processed on Report Card software and the following parameters were obtained: RV end-diastolic volume (EDV), end-systolic volume (ESV), stroke volume (SV), ejection fraction (EF), and myocardial mass (MM). Except for EF, all of the above parameters were normalized to body surface area (BSA). PC-MRI images were post-processed on Report Card software, peak velocity and distensibility of main pulmonary artery (MPA) could also be obtained. Student *t* test was employed for statistical assessment.

**Results:**

Compared with controls, RV EDV, ESV and MM index in PH patients were significantly increased (*P* < 0.01), EF was significantly impaired (*P* < 0.01), peak velocity and distensibility of MPA were significantly lower (*P* < 0.01). SV index between the two groups had no significant difference (*P* > 0.05).

**Conclusion:**

Cardiac MRI is of great value in the assessment of RV function and MPA hemodynamic parameters in patients with PH.

肺动脉高压（pulmonary hypertension, PH）是一种血液动力学和病理生理学状态，可见于多种临床疾病中，其特征是肺血管的增殖和重构^[1]^，使肺血管床阻力增加、肺动脉压力升高，进而右心室重构、功能下降，最终导致进行性右心功能衰竭而死亡^[2, 3]^。因而准确、无创地监测患者右心功能及肺动脉血液动力学改变对于了解PH患者病情进展程度、判断预后和指导治疗都具有重要意义。

目前，心脏MRI（cardiac magnetic resonance imaging, CMRI）已成为无创性评价右心室结构及功能的理想方法，该法受右心室形态不规则的影响较小，准确性高，可重复性强^[4, 5]^。本研究旨在运用CMRI技术，比较国人PH患者与健康志愿者之间在右心功能和肺动脉主干血液动力学参数上存在的差异，为进一步探究CMRI在PH中的应用提供依据。

## 1 资料与方法

### 1.1 成像设备

GE 1.5T Twin-speed Infinity with Excite Ⅱ超导型磁共振扫描仪，8通道心脏相控阵线圈，呼吸门控及心电门控。

### 1.2 研究对象

按照2009欧洲心脏病学会肺动脉高压诊断和治疗指南的标准^[1]^，2008年1月至2012年3月期间，在我院经右心导管确诊的PH患者（静息状态下肺动脉平均压≥25 mmHg，1 mmHg=0.133 kPa）共25例，男性6例，女性19例，年龄（38.0±10.5）岁，心率（78.0±9.2）次；其中5例近期诊断为PH，未接受药物治疗，20例接受正规治疗，治疗时间为6个月-47个月。本组25例患者中慢性血栓栓塞性肺动脉高压（chronic thromboembolic pulmonary hypertension, CTEPH）5例，动脉型肺动脉高压（pulmonary arterial hypertension, PAH）20例，均排除冠心病、心脏瓣膜病、慢性阻塞性肺疾病（chronic obstructive pulmonary disease, COPD）等其它心肺疾病。健康志愿者30例，男性7例，女性23例，年龄（36.0±8.9）岁，心率（72.4±7.1）次，年龄、性别与PH组相匹配，心率、血压均在正常范围，无心、肺疾病史。所有受试者同意参加本研究并签署知情同意书。

### 1.3 检查序列及成像参数

采用二维快速稳态进动采集序列（fast imaging employing steady-state acquisition, FIESTA）获得左、右心室二腔心、四腔心及短轴位电影CMRI图像。根据受试者心率不同，每层图像采集期间需屏气约9 s-13 s。FIESTA序列短轴位成像参数：TR/TE min full/min full，翻转角45°，带宽125 kHz，FOV 35 cm×35 cm，矩阵224×224，扫描层厚/间隔8/0 mm，NEX 1，包括全部右心室需获9层-13层图像，每层扫描时相为20。

采用Fast Cine相位对比（phase-contrast, PC）序列进行主肺动脉（main pulmonary artery, MPA）血流测量，回顾性心电门控触发分段K空间采集成像，屏气扫描。定位方法：在轴位图像上，取平行于MPA走行方向，得到MPA长轴图，在长轴图肺动脉瓣上方1.5 cm-2 cm处取垂直于MPA长轴方向行单层多时相扫描，得到相应的幅度图（magnitude image）和相位图（phase-map image）（图 1）。Fast Cine PC序列成像参数：TR/TE自动选择最小重复时间/min full，翻转角20°，带宽31.25 kHz，FOV 35 cm×35 cm，矩阵256×256，NEX 1，扫描时相为30。流速编码方向为SLICE，速度编码值为150 cm/s。全部检查时间约30 min。

**1:**
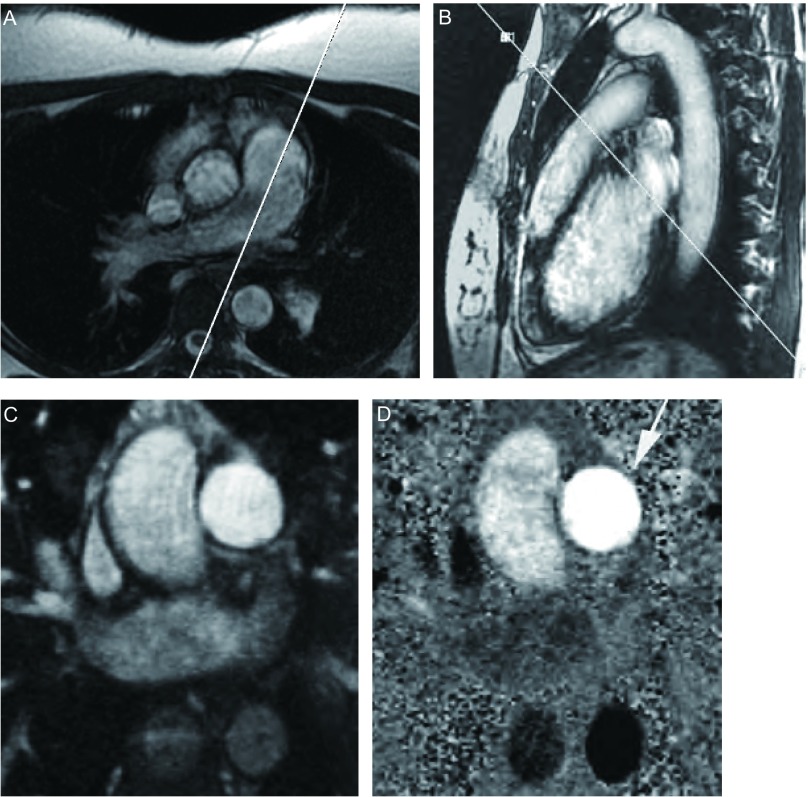
MPA的PC-MRI血流测量。在轴位图像上取平行于MPA走行方向（A）扫描得到MPA长轴图（B），在长轴图肺动脉瓣上方1.5 cm-2 cm处取垂直于MPA长轴方向扫描，得到相应的幅度图（C）和相位图（D）。MPA（箭）血流方向与流速编码方向一致，显示为亮信号。 Flow measurement in MPA by PC-MRI. The longaxis image of MPA (B) was prescribed parallel to the MPA long-axis in the axial image (A). An image plane was positioned perpendicular to the long-axis image of MPA, 1.5 cm-2 cm above the level of the pulmonary valve, resulting in magnitude (C) and phase-map images (D). Flow direction in MPA was the same with the velocity encoding direction, flow in MPA (arrow) was displayed as white.

### 1.4 图像分析

将所有数据传至GE AW 4.3 MRI工作站，应用Report Card软件进行数据分析。

在短轴位FIESTA电影图像中进行右心室功能分析。舒张末期与收缩末期时相分别为心室达最大容积与最小容积的时相。手动描记收缩末期与舒张末期所有图像的右心室心内膜与心外膜轮廓（图 2）。右心室容积包括右心室流出道容积。乳头肌和小梁作为心室腔的一部分，其质量不包括在右心室质量的计算内。软件自动计算出舒张末期容积（end-diastolic volume, EDV）、收缩末期容积（end-systolic volume, ESV）、搏出量（stroke volume, SV）、射血分数（ejection fraction, EF）及心脏舒张末期心外膜容积。舒张末期心室肌质量计算运用以下公式：心肌质量（myocardial mass, MM）=（心外膜容积-心内膜容积）×1.05。EDV、ESV、SV及MM均除以体表面积（body surface area, BSA）后，即记为EDV指数、ESV指数、SV指数、MM指数，再作统计学分析。

**2:**
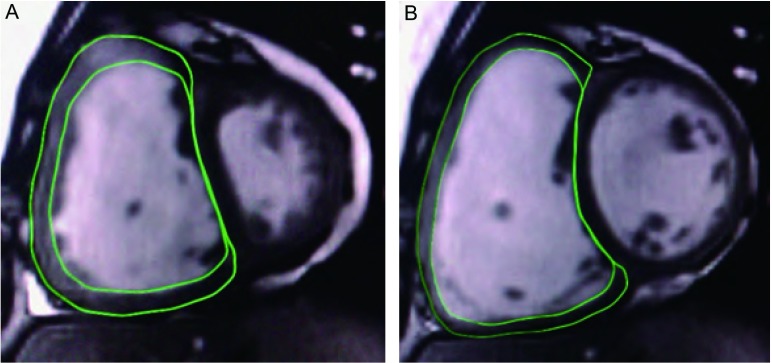
FIESTA序列心脏短轴位图。A为心室收缩末期；B为心室舒张末期。图中显示出描记右心室心内膜和心外膜轮廓的方法。 FIESTA short-axis images covering right and left ventricles. A: RV end-systolic phase; B: RV enddiastolic phase. The images showed RV endocardial and epicardial contours.

血液动力学参数测量方法如下：在轴位幅度图中，沿MPA血管轮廓内缘手动描记，勾画出感兴趣区（region of interest, ROI），软件自动在一个心动周期各个时相追踪ROI，并将ROI位置复制到相应相位图，如生成的血管轮廓有偏离则手动调整。确定ROI后，软件计算出MPA在一个心动周期的正向峰值流速、最大截面积和最小截面积，MPA顺应性（distensibility）= [（最大截面积-最小截面积)/最小截面积]×100。

### 1.5 统计学分析

采用SPSS 17.0统计软件对数据进行统计学分析，计量资料以Mean±SD表示。对测得的PH患者与健康志愿者各指标采用两独立样本*t*检验进行差异分析。*P* < 0.05为差异具有统计学意义。

## 2 结果

55例受试者均顺利完成CMRI检查。全部图像清晰，无伪影。PH组与健康对照组相比，右心室EDV、ESV及MM指数均明显高于后者（*P* < 0.01），EF明显低于后者（*P* < 0.01），PH组SV指数与对照组无明显差异（*P* > 0.05），PH组MPA峰值流速及顺应性均明显低于对照组（*P* < 0.01），详见表 1。

**1:** PH组与对照组右心功能及MPA血液动力学参数比较（Mean±SD） Comparison of RV function and MPA hemodynamic parameters between PH and control group (Mean±SD)

Parameters	Control group	PH group	*t*	*P*
No. of patients (male/female)	30 (7/23)	25 (6/19)	-	-
EDV index (mL/m^2^)	74.0±8.5	112.2±30.6	6.06	< 0.001
ESV index (mL/m^2^)	31.6±5.0	70.4±25.0	7.63	< 0.001
SV index (mL/m^2^)	42.3±6.9	41.3±9.3	0.46	0.730
MM index (g/m^2^)	24.4±3.7	40.0±7.6	9.37	< 0.001
EF (%)	57.2±5.4	38.2±8.6	10.21	< 0.001
MPA peak velocity (cm/s)	82.3±16.0	62.4±11.2	5.39	< 0.001
MPA distensibility (%)	46.7±15.2	20.1±6.9	8.58	< 0.001
EDV: end-diastolic volume; ESV: end-systolic volume; SV: stroke volume; MM: myocardial mass; EF: ejection fraction; MPA: main pulmonary artery.				

## 3 讨论

PH是肺动脉压力持续高于正常的病理状态，可见于多种临床疾病状态中，近年来发病率呈上升趋势。根据2009欧洲心脏病学会发布的指南，可将PH分为6组临床类型，每组类型具有不同的病理学、病理生物学、遗传学、流行病学和致病危险因素等方面的特征，每一组类型又见于多种临床疾病。目前缺乏可比较的流行病学数据^[1]^。文献报道PAH（第1组）发病率在欧洲大约为（15-50）人/百万人^[1, 6]^，左心疾病所致的PH（第2组）几乎见于所有伴明显症状的二尖瓣疾病患者^[1, 7]^，肺疾病所致的PH（第3组）在进展性COPD患者中的发病率可大于50%，CTEPH（第4组）可见于0.5%-2%曾患急性肺栓塞的患者，其它两组（肺静脉闭塞性疾病和/或肺毛细血管瘤病、原因不明和多种机制所致的PH）流行病学数据缺乏^[1]^。本组PH病例包括了PAH和CTEPH两组类型。PH诊断标准为静息状态下经右心导管测量的肺动脉平均压≥25 mmHg^[1]^。PH患者右心后负荷增加，右心室输出量逐渐下降、右心房压力逐渐升高造成进行性右心功能衰竭，多数患者预后差。因此，准确、无创评估和监测PH患者右心功能受损程度，对研究和制定有效的治疗措施、提高生存质量和改善预后都尤为重要。

超声心动是临床筛查PH最常用的检查方法，但该法受检查者的经验、受试部位（胸骨位置或严重的肺气肿）声窗限制的影响较大。由于右心室形态不规则，加上肌小梁的影响，超声心动模拟柱形测量右心室容积等数据并不准确。右心导管法是确诊PH的金标准，但此方法是有创检查，并且存在电离辐射、受三尖瓣反流影响误差较大等缺点。因而以上两种方法不是评估和随访监测PH患者右心功能的理想方法。随着平衡稳态自由进动（balanced steady-state free precession, bSSFP）序列的出现和完善，CMRI解决了舒张期图像中血流边界不易分辨的问题，运动伪影少，且能获得更高的信噪比；与此同时，硬件的发展使CMRI图像的采集时间明显缩短，受试者所需屏气时间亦随之缩短，这使得CMRI能被更广泛地应用于临床及实验研究。如今，CMRI已被认为是评价右心室结构及功能的理想工具，除了可以准确测量右心室容积、SV、EF、MM等参数外，还能提供大血管的解剖结构、血流方向、流速及流量等信息^[8, 9]^。

本组PH患者右心室EDV指数与ESV指数较正常者增大，代表右心室的扩张；患者右心EF降低说明心室收缩功能受损；MM指数明显增加提示患者心室肌代偿性肥厚。上述结果与近期国外一些研究结果^[10, 11]^相近。对于PH患者，肺动脉阻力增加使右心室压力负荷增大，进而右心室代偿性肥大，右心室、心房扩张及三尖瓣反流。上述情况如不加以正规、有效的干预治疗，则右心室功能会因失代偿而进行性下降，并可发生右心衰竭甚至心源性猝死^[12]^。本研究中，PH组右心室SV指数与正常组无明显差异，提示本组PH患者右心代偿性肥大以及右心的扩张尚可维持正常的搏出量。虽然PH直接影响右心室的形态及功能，但左心室功能也会发生变化。Gan等^[13]^认为右心室形态与功能变化会通过室间隔影响左心室充盈，进而降低左心室搏出量；Kuehne等^[14]^研究6例特发性肺动脉高压患者发现，在疾病早期右心室功能无明显降低时，左心室搏出量即有下降。PH患者左心功能变化的规律尚有待今后继续探究。

肺动脉顺应性由动脉壁的主要组成成分-弹性纤维和胶原纤维决定。本研究中PH组MPA顺应性明显低于正常组，与最近Kang等^[15]^研究结果相近，其原因可能是肺动脉压力升高时血管壁受牵拉，可扩张的胶原纤维减少，使管壁变僵硬，顺应性随之降低。肺动脉顺应性对PH患者肺动脉压力高低的反映及其对评估患者预后的价值，有待于进一步研究。此外，本组PH患者MPA峰值血流速度明显低于健康志愿者，与Ley等^[16]^研究结果相近，反映出PH患者肺动脉内血流阻力明显高于正常状态，使整个肺循环速度变慢，MPA峰值血流速度亦降低。Sanz等^[17]^对42例PAH患者进行了CMRI检查，结果显示MPA峰值流速与肺动脉收缩压、肺血管阻力指数呈负相关，支持本研究的结论。此外，MPA管径增粗也可能是导致自身血流速度下降的原因之一。

CMRI亦存在一些不足之处：操作较复杂，检查时间相对较长，且需要患者很好地配合屏气，因而不适用于病情较重的患者；有幽闭恐惧症、体内带有磁性金属植入物的患者不适用于本法。另外，多种原因引起的伪影有时会影响图像质量。

总之，CMRI能较准确地为临床提供PH患者右心功能及肺动脉血液动力学参数的信息，同时也将在探究PH发病机制、研究和制定有效的治疗方案、随访疗效和监测疾病发展过程及转归中发挥重要的作用。
